# Characterization of THSD7A-antibodies not binding to glomerular THSD7A in a patient with diabetes mellitus but no membranous nephropathy

**DOI:** 10.1038/s41598-021-94921-y

**Published:** 2021-08-10

**Authors:** Linda Reinhard, Cindy Thomas, Maya Machalitza, Erik Lattwein, Lothar S. Weiss, Jan Vitu, Thorsten Wiech, Rolf A. K. Stahl, Elion Hoxha

**Affiliations:** 1 III. Department of Medicine, University Medical Center Hamburg-Eppendorf, Martinistrasse 52, 20246 Hamburg, Germany; 2 Institute of Experimental Immunology, EUROIMMUN AG, Lübeck, Germany; 3 Medizinisches Versorgungszentrum Hamburg-Sinstorf der MVZ gGmbH der PHV, Hamburg, Germany; 4 Institute of Pathology, Section Nephropathology, University Medical Center Hamburg-Eppendorf, Martinistrasse 52, 20246 Hamburg, Germany

**Keywords:** Biological techniques, Immunology, Biomarkers, Diseases, Health care, Nephrology

## Abstract

Membranous nephropathy (MN) is an autoimmune disease caused by autoantibodies against the podocyte antigens phospholipase A_2_ receptor 1 (PLA_2_R1) and thrombospondin type 1 domain containing protein 7A (THSD7A) in 80% and 2–3% of patients, respectively. THSD7A antibodies are considered to be pathogenic and highly specific for MN patients. Using an indirect immunofluorescence test (IIFT) we detected THSD7A-antibodies (titre 1:10) in the serum of a patient with high proteinuria who, however, in the kidney biopsy was diagnosed with diabetic nephropathy and MN was excluded as a possible cause of proteinuria. Different immunofluorescence assays and Western blot techniques using recombinant THSD7A (rTHSD7A) or THSD7A from different human tissues revealed that the circulating THSD7A-autoantibodies were only of the IgG3 subclass. The patient serum reacted exclusively with rTHSD7A and only when the antigen was present in reducing Western blot conditions, or on formaldehyde-fixed cells for the IIFT. Our findings show for the first time the existence of circulating THSD7A-antibodies recognizing denatured/reduced rTHSD7A, which do not react with glomerular THSD7A in vivo and are thus presumptively non-pathogenic. As a consequence, kidney biopsy or Western blot analyses of THSD7A under non-reducing conditions should be performed to confirm the diagnosis of THSD7A-associated MN, especially in cases with low THSD7A-antibody levels in the IIFT.

## Introduction

Membranous nephropathy (MN) is an autoimmune disease in which circulating autoantibodies bind to target antigens expressed on the surface of glomerular podocytes. Phospholipase A_2_ receptor 1 (PLA_2_R1) and thrombospondin type 1 domain containing protein 7A (THSD7A) are known target antigens, and autoantibodies are found in 80% and 2–3% of MN patients, respectively^1,2^. MN is diagnosed based on immunohistochemical and electron microscopy analyses of renal biopsies, which typically show subepithelial IgG deposits and an enhanced staining for the target antigens within the glomerulus^2,3^. The detection of autoantibodies in patient sera has become a standard technique in the diagnosis of MN^4^. A variety of techniques are being used to identify and quantify these autoantibodies in patient sera, including ELISAs, indirect immunofluorescence tests (IIFT), Western blot techniques and a chemiluminescence immunoassay (ChLIA)^1,2,5–11^. For PLA_2_R1 it was shown that the sensitivity of the methods increases in the order from ELISA, IIFT to Western blot^6^. Similarly, Western blot is also more sensitive than IIFT for the detection of THSD7A-antibodies^5^.

With the identification of the PLA_2_R1 and THSD7A as autoantigens, it is possible to make a pathogenesis-based diagnosis in the large majority of MN patients^12^. Since the detection of autoantibodies in the blood is highly specific for the diagnosis of MN, the question has been raised, whether a diagnostic kidney biopsy should still be performed in nephrotic patients who are positive for PLA_2_R1 or THSD7A-antibodies^13,14^.

Here we describe for the first time the presence of circulating THSD7A-antibodies in a patient with proteinuria, but no MN. Kidney biopsy excluded MN and showed a diabetic nephropathy as the cause of proteinuria. A biochemical characterization of these circulating THSD7A-antibodies was performed in order to elucidate these discordant diagnostic results and investigate their clinical relevance.

## Results

### Clinical presentation

A 66 years old male patient with a known history of diabetes mellitus type I presented with oedema of the lower limbs and proteinuria of 1.4 g/g creatinine. His serum creatinine was 1.26 mg/dl and eGFR was 59 ml/min/1.73 m^2^. His blood pressure was well controlled under medication with an ACE-inhibitor. The medical history of the patient was otherwise unremarkable, in particular there were no signs of a secondary cause of MN i.e. tumour, lupus erythematosus, infection, etc.

**Figure 1:**
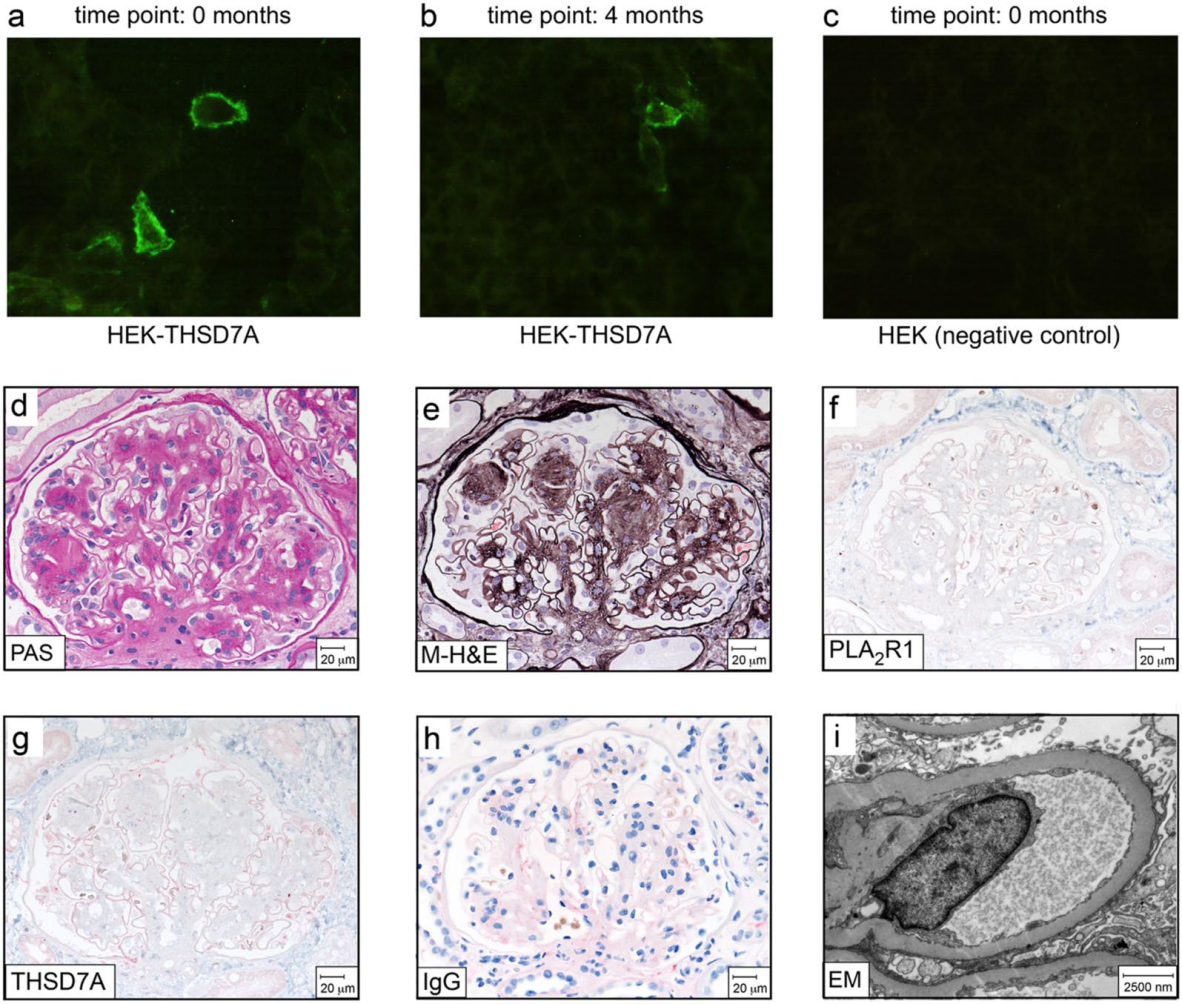
Findings in patient serum and renal biopsy. (**a**–**c**) Representative photographs of the results of the IIFT assay. (**a**) At the time of the initial presentation of the patient (0 months), THSD7A-antibodies are detectable in the IIFT analysis at a titre of 1:10. (**b**) The same level of positivity is observed after 4 months. (**c**) The negative control HEK cells, which are transfected with an empty expression vector do not express THSD7A and are analysed simultaneously with the patient serum “0 months”, show no detectable fluorescence. Incubation of the THSD7A-expressing HEK cells with a healthy serum showed no signal (not shown). The IIFT results were evaluated blinded in two different laboratories, in each lab by at least two different investigators. (**d**) PAS staining and (**e**) methenamine silver-H&E (M-H&E) staining reveal lesions typical for diabetic nephropathy, i.e. nodular glomerulosclerosis. Immunohistochemical staining for (**f**) PLA_2_R1, (**g**) THSD7A and (**h**) IgG show no signs of MN. (**i**) By electron microscopy no electron dense subepithelial deposits, which are characteristic for MN, are detectable.

Because of the proteinuria, the serum of the patient was tested for the presence of PLA_2_R1- and THSD7A-antibodies (time point: 0 months). The serum was negative for PLA_2_R1-antibody by ELISA, IIFT and Western blot, while a low titre THSD7A-antibody (1:10) was detected by IIFT (Fig. 1a). For comparison, in a prospective cohort of 57 THSD7A-antibody positive MN patients, which in large parts has been described before^5,15^, 11 patients were found to have a THSD7A-antibody titre of less than 1:100 by IIFT (data not shown).

Since moderate to high level proteinuria is a clinical hallmark in about 20% of patients with diabetic nephropathy but may also be caused by MN, the measurement of serum THSD7A-antibody titre was repeated four months after the first testing. The same results were obtained with low titre THSD7A-antibody (1:10) and no PLA_2_R1-antibody (Fig. 1b,c). Since the renal function of the patient had worsened (serum creatinine 1.6 mg/dl), a renal biopsy was performed 7 months after the first clinical presentation. The work-up revealed the diagnosis of diabetic nephropathy and excluded MN (Fig. 1d–i). There were no detectable IgG depositions, no enhanced staining for THSD7A or PLA_2_R1 and no electron dense deposits visible by electron microscopy. The biopsy showed a typical pattern of diabetic nephropathy.

### Western blot analyses

Because of the discrepant findings in the serology and kidney biopsy, a third methodology was used to evaluate if THSD7A-antibodies are detectable in the sera of the patient. For the detection of THSD7A-antibodies in patient sera, the standard procedure is a Western blot under non-reducing conditions, which allows conformation sensitive epitopes of the antigen to remain intact^2,16^.

**Figure 2:**
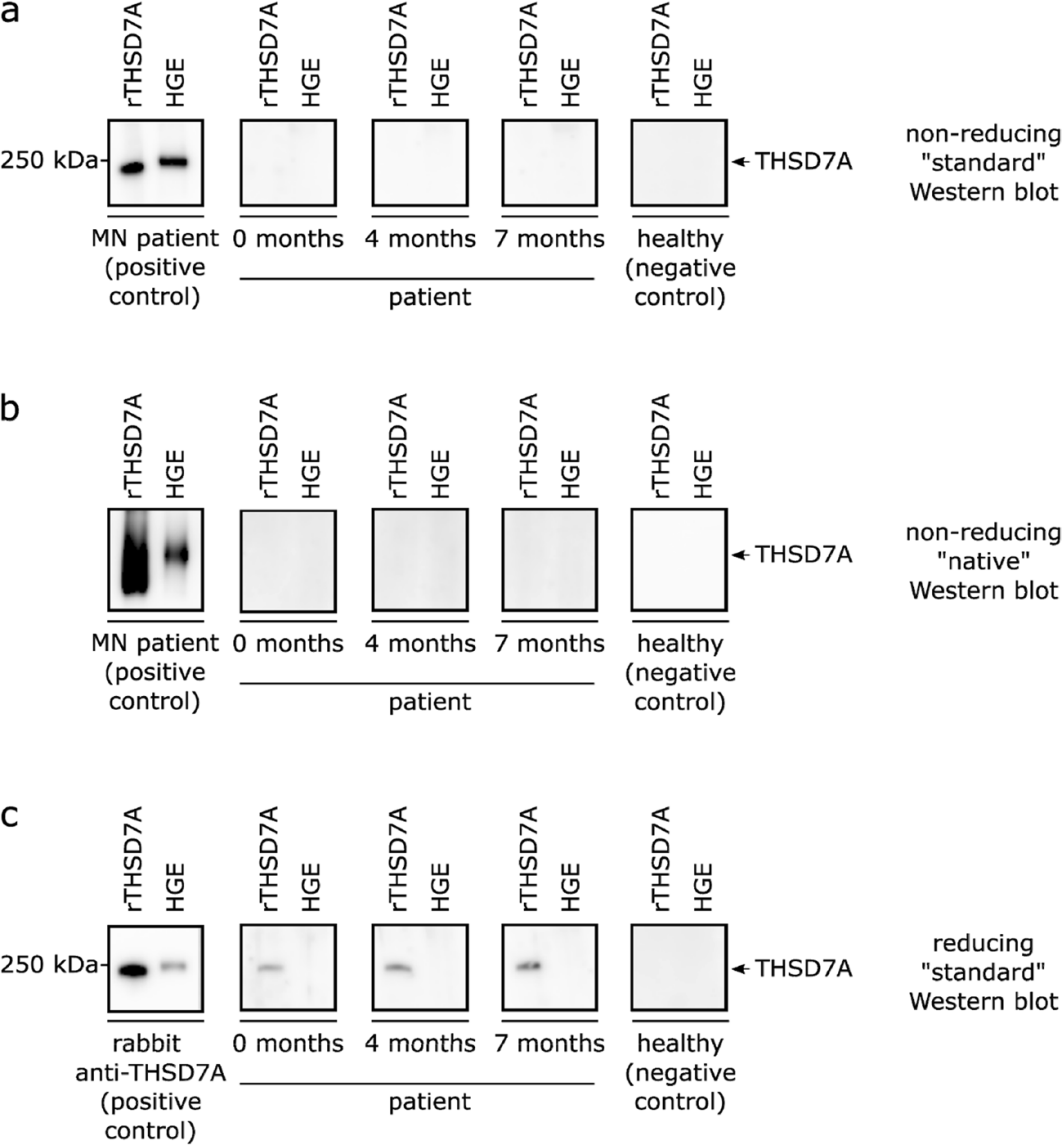
Results of Western blot analyses. In Western blot analyses serum of the patient was used as primary antibody. The Western blots, which were performed under (**a**) non-reducing, “standard” conditions (SDS present); and (**b**) non-reducing, “native” conditions (SDS absent) do not give any detectable THSD7A-specific signal. For the positive control in A and B, serum from a THSD7A-antibody positive MN patient was used. (**c**) Western blot analysis under reducing, “standard” conditions (SDS present) gave a Western blot signal at the expected height for rTHSD7A, but not for HGE. Also for lung tissue extract no signal was obtained (not shown). For the positive control, rabbit anti-human THSD7A was used. For all three analyses, serum from a healthy donor served as negative control.

Therefore, the Western blot analysis using recombinant THSD7A (rTHSD7A) as well as human glomerular extracts (HGE) was performed under non-reducing conditions. No detectable band appeared when the serum of the patient was used as a primary antibody, even not after prolonged exposure time (Fig. 2a; Supplemental Figure 1a). Even though the Western blot was performed under “standard”, non-reducing conditions, the conditions can still be considered as being harsh, since the detergent SDS is present and samples are heated to 95 °C prior to loading. In order to analyse if the patient exhibits THSD7A-antibodies, which recognize conformation sensitive epitopes that are destroyed under these experimental conditions, a blue native polyacrylamide gel electrophoresis (BN-PAGE) was performed as an electrophoretic step to separate the proteins in their native conformation. Also this “native” Western blot analysis did not give any THSD7A specific signal (Fig. 2b; Supplemental Figure 1b).

As a next step, a “standard” Western blot analysis was performed under reducing conditions (Fig. 2c; Supplemental Figure 1c). Surprisingly, the patient’s serum gave a specific signal at a molecular weight corresponding to THSD7A, but only when rTHSD7A was used as antigen, and not when THSD7A from human glomerular extracts or lung tissue extracts were used^15^. IgG subclass analysis revealed that these antibodies solely belong to the IgG3 subclass (Supplemental Figure 2a). Analysis of sera from 8 patients with THSD7A-associated MN showed THSD7A-antibody positivity in all cases by non-reducing Western blot and identified 3 cases with a positive THSD7A signal detectable under reducing conditions. For all three MN patients, these antibodies belong to the IgG4 subclass, whereas one MN patient additionally has antibodies of the IgG3 subclass (Supplemental Figure 2b–d). In contrast, sera from a control cohort (8 PLA_2_R1-antibody positive MN patients; 3 patients with minimal change disease and 3 patients with IgA nephropathy) did not give any positive signal.

Next, histidine-tagged THSD7A was recombinantly produced in HEK cells (extracellular domain) and purified using immobilized metal ion affinity chromatography (IMAC) under denatured conditions (elution with urea containing buffer) to be used in the Western blot analysis. In this experiment, the serum of the patient again gave a clear Western blot signal, indicating that indeed THSD7A is the protein being recognized by the serum. Deglycosylation of the rTHSD7A did not result in abolishment of the signal, showing that the recognition is not dependent on the glycosylation status of the protein (Supplemental Figures 3a, 4a). Noteworthy, the serum of the patient and serum from a control patient with THSD7A-associated MN gave the same recognition pattern when deglycosylated THSD7A was used as antigen. We found no evidence for significant differences in the glycosylation patterns of circulating IgG3 between our patient with diabetic nephropathy, a healthy control and a patient with THSD7A-associated MN, since serum IgG3 gave a Western blot signal at the same height in all cases, indicating similar molecular weight (Supplemental Figures 3b, 4b).

### Co-staining IIFT experiments

**Figure 3:**
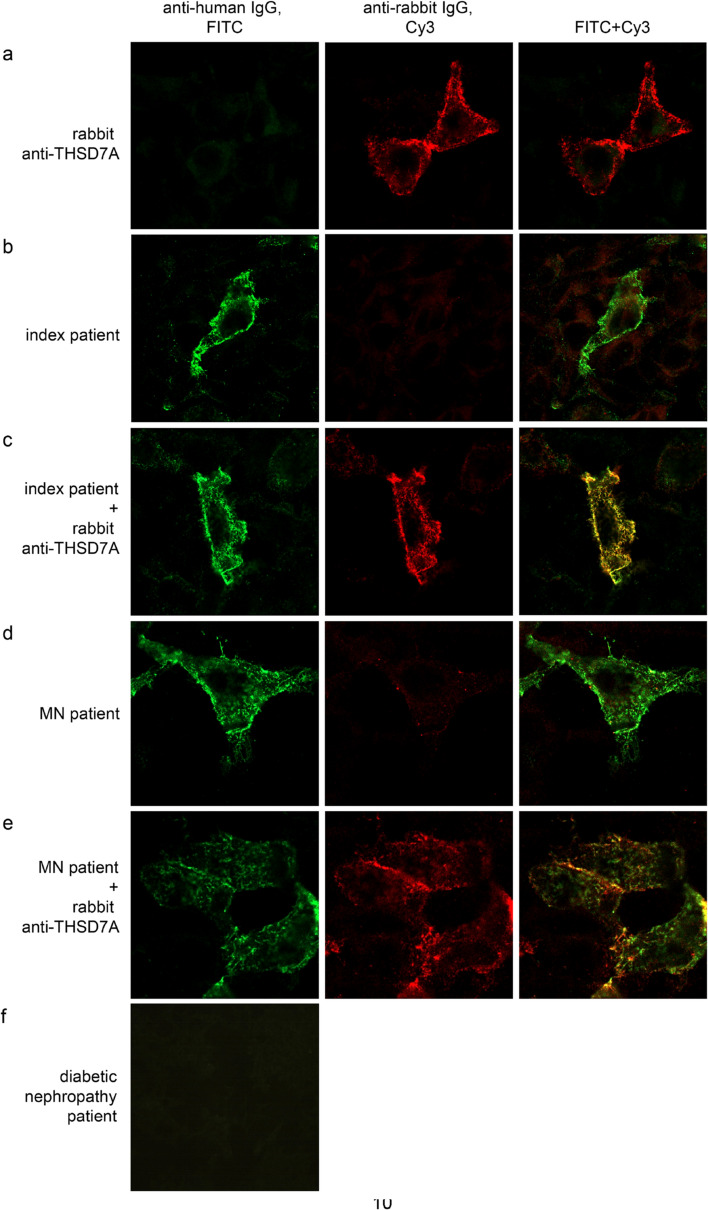
Immunofluorescence co-staining. Representative photographs of THSD7A-expressing HEK cells co-stained with FITC-coupled anti-human IgG (in green) and Cy3-coupled anti-rabbit IgG (in red). (**a**–**e**): as primary antibody the following was used: (**a**) a commercial rabbit anti-THSD7A; (**b**) serum from the presented patient case; (**c**) a combination of rabbit anti-THSD7A with serum from the patient case; (**d**) serum from a patient with THSD7A-associated MN and (**e**) a combination of rabbit anti-THSD7A with serum from a patient with THSD7A-associated MN. Co-staining experiments reveal that serum of the patient case (**c**) and a patient with THSD7A-associated MN (**e**) co-localize with the THSD7A-specific rabbit antibody, showing that both sera specifically recognize THSD7A on the THSD7A-expressing HEK cells. (**f**) Representative photograph of THSD7A-expressing HEK cells assayed with serum from a patient with diabetic nephropathy but no membranous nephropathy and visualized with FITC-coupled anti-human IgG.

The Western blot results provide evidence that autoantibodies in the serum of the patient recognize one or more THSD7A epitopes, which are present only under denaturing and reducing conditions in the recombinant but not the naturally occurring THSD7A. In order to confirm this finding, we performed IIFT co-staining experiments using a commercial rabbit THSD7A-antibody and serum from the patient (Fig. 3a–c). The commercial rabbit THSD7A-antibody and autoantibodies from the patient serum perfectly co-localized to THSD7A expressing HEK cells, again providing evidence that the antigen bound by both antibodies is the same.

As a positive control, we performed a co-staining of THSD7A-expressing HEK cells using the commercial rabbit THSD7A-antibody and serum from a THSD7A-antibody positive patient with MN. We again found a perfect co-localization of the two antibodies (Fig. 3d,e). Taken together, these results confirm the Western blot results indicating that the serum is able to recognize the THSD7A expressed by the HEK cells. As a negative control cohort, we analysed 25 patients with diabetic nephropathy but no membranous nephropathy using the standard IIFT. In the blood of these patients, no THSD7A-antibodies were detectable (Fig. 3f).

Noteworthy, the THSD7A proteins which give the positive signals in Western blot analyses and on the IIFT originate from HEK cells recombinantly expressing the protein. It needs to be considered that for fixation the HEK cells on the IIFT biochip are pre-treated with formaldehyde, possibly with a similar effect as the denaturing and reducing procedures in the Western blot experiment. In contrast, THSD7A derived from human tissues did not bind at any experimental condition to the autoantibodies in the serum of the patient. Therefore, we performed an in-house developed, live cell-based assay (live CBA) using HEK cells, which express the full-length human rTHSD7A on the surface, but are not fixed during the experimental procedures. Under these conditions, the patient serum did not react with rTHSD7A (Supplemental Figure 5), again confirming the results of the Western blot experiments, indicating that the serum is able to recognize only denatured/reduced rTHSD7A, but not the native form of the protein.

## Discussion

The detection of PLA_2_R1- or THSD7A-antibodies in the serum of patients is very specific for the diagnosis of MN. Therefore, it is discussed, whether or not a kidney biopsy is needed to make the diagnosis MN in nephrotic patients who are tested positive for these antibodies in the serum^13,14^. Here, we report the first case of a patient, who was tested positive for THSD7A-antibody by IIFT, but no MN was detected when the renal tissue was histomorphologically analysed. Instead the patient was found to have a diabetic nephropathy as the consequence of his type I diabetes mellitus.

Our study aimed to characterize these circulating THSD7A-antibodies and better understand these discrepant results between the serum measurement of THSD7A-antibodies and the morphologic findings in the kidney biopsy. The most sensitive method for the detection of circulating THSD7A-antibody was shown to be the Western blot technique under non-reducing conditions^5^. In the sera of the patient no THSD7A-antibody could be detected using non-reducing “standard” or “native” Western blot techniques. These results suggest that the serum of the patient does not recognize correctly folded THSD7A, as one would expect the protein to be present in the body. These findings are supported by the kidney biopsy, which showed neither deposition of human IgG in glomeruli, nor an increased staining for THSD7A or electron-dense immune deposits, which are typical for THSD7A-associated MN.

To our surprise, the serum of the patient was capable of recognizing rTHSD7A under reducing conditions in the Western blot analysis. Under these destructive conditions, an epitope, which is hidden in the correctly folded protein, can be recognized by antibodies of the IgG3 subclass. The immunofluorescence assay results confirmed these observations and the serum reacted to rTHSD7A only on formalin-fixed HEK cells, but not when a live-cell assay was performed. The IIFT has previously been tested using a control cohort of 112 patients with glomerular diseases other than membranous nephropathy and none of them gave a positive signal^5^. Moreover, 25 patients with diabetic nephropathy and no membranous nephropathy analysed in this study were all negative in the IIFT. Noteworthy, THSD7A present in human glomerular extract or lung tissue extract is not recognized by the serum. The recognition might therefore originate from a modification of the recombinant protein, such as a post-translational modification, which is not present in the in vivo situation. This interpretation is supported by the findings in the kidney biopsy. Taken together, it is likely that the observed in vitro reactivity of the patient serum does not occur within the human body.

Interestingly, sera from three out of eight patients with THSD7A-associated MN also showed a THSD7A-Western blot signal under reducing conditions. In contrast, none of the analysed control sera (PLA_2_R1-associated MN, minimal change disease, IgA nephropathy, healthy control) generated a similar Western blot signal. Noteworthy, the three patient sera exhibit a high THSD7A-antibody titre and the corresponding antibody subclass is predominantly IgG4. It is possible, that in these patients THSD7A-antibodies recognize different epitopes, some of which might be non-conformational. However, we cannot exclude, that refolding of THSD7A proteins may take place during the Western blot experiment and account for this finding. We found no evidence suggesting that an altered glycosylation pattern of the antigen or the antibody might be responsible for our findings.

The possibility of a false positive test always has to be considered in clinical laboratory routine. In our view, there is no doubt that the patient did not have MN. However, the patient has THSD7A-antibodies in circulation, which are most probably non-pathogenic in relation to causing MN. Nevertheless, two independent, experienced laboratories generated the same IIFT findings, showing that most likely such cases will be falsely characterized in the clinical routine. This, of course, has important clinical implications since treatment of MN includes medications with potential toxicity and therefore making the exact diagnosis is essential. Further, our results demonstrate the need of applying different techniques to make a safe diagnosis of MN. Performing experiments with native antigens would be an alternative, which, however, is not suited to clinical routine. This report implies that, in cases when the diagnosis of THSD7A-associated MN is solely based on serology, performing a native antigen assay is needed. In order to make an exact diagnosis a kidney biopsy is needed, especially if THSD7A-antibody levels are low or patients present with an untypical clinical manifestation.

## Methods

### Measurement of PLA_2_R1 and THSD7A antibodies

Three serum samples were collected from the patient over a follow-up period of seven months. Sera of the patient were assayed for the presence of PLA_2_R1-antibodies by a PLA_2_R1-specific ELISA (EUROIMMUN; #EA1254G)^6^. Serum samples were then analysed for the presence of PLA_2_R1- and THSD7A-antibodies using an indirect immunofluorescence test (IIFT)^5^, following the manufacturer protocol (EUROIMMUN; #FA1254-1). Serum dilutions of 1:10 and 1:100 in PBS supplemented with 0.2% (v/v) Tween-20 were used in the analysis. All analyses were performed in two independent laboratories. In each of the laboratories, at least two blinded investigators analysed the results independently.

Sera used for control experiments have been reported before^15,17^. The study was approved by the local ethics committee of the chamber of physicians in Hamburg and conducted in accordance with the ethical principles stated by the Declaration of Helsinki. An informed consent was obtained from the patients.

### Histology and immunohistochemistry

PAS staining and combined methenamine silver-hematoxylin and eosin (M-H&E) staining was performed following standard protocols. Immunohistochemical staining for PLA_2_R1, THSD7A and IgG were performed as previously reported^14^.

### Western blot analyses

Human glomerular extract (HGE) and recombinant THSD7A (rTHSD7A) were prepared as previously reported^2,16^. For analysis of IgG3 in human sera, the sera were pre-diluted 1:1000 in PBS. The proteins were separated by standard SDS-PAGE (non-reducing and reducing conditions) and by blue native PAGE^18^. Subsequent Western blot analysis was performed as previously published, using the serum as primary antibody at a 1:100 dilution in 0.5% (w/v) milk in PBS supplemented with 0.1% Tween-20 (PBS-T)^2^. As secondary antibody, HRP-conjugated mouse anti-human IgG1 Fc (SouthernBiotech; #9054-05; 1:5000 in a blocking buffer of 3.5%(w/v) milk in PBS-T), anti-human IgG2 Fc (SouthernBiotech; #9060-05; 1:5000 in blocking buffer), anti-human IgG3 Hinge (SouthernBiotech; #9210-05; 1:10,000 in blocking buffer), anti-human IgG4 Fc (SouthernBiotech; #9200-05; 1:10,000 in blocking buffer), or IgG Fc (SouthernBiotech; #9040-05; 1:20,000 in blocking buffer) were used. For the positive control under reducing Western blot conditions, rabbit anti-human THSD7A (Sigma Aldrich; #HPA000923-100UL; 1:1000 dilution in 0.5%(w/v) milk in PBS-T) was used as primary antibody, followed by detection with HRP-coupled goat anti-rabbit IgG (Sigma Aldrich; #A9169; 1:20,000 dilution in blocking buffer).

### Deglycosylation

rTHSD7A in 100 mM Tris–HCl pH 8.0, 20% glycerol and 50 mM NaCl supplemented with 1 × complete™, EDTA-free protease inhibitor cocktail was incubated with either *N*-glycosidase F, *O*-glycosidase or Neuraminidase (all from Sigma Aldrich), or a combination of the three. The samples were incubated for 1 h at 37 °C under gentle agitation. The samples were directly used for Western blot analysis under non-reducing conditions.

### Co-staining in IIFT

For co-staining experiments, the commercially available IIFT was used (EUROIMMUN; #FA1254-1), with a slightly modified protocol. Serum samples were diluted 1:10 in PBS supplemented with 0.2% (v/v) Tween-20. For the co-staining conditions, rabbit anti-human THSD7A (Sigma Aldrich; #HPA000923-100UL) was added directly to the serum dilution in a 1:100 dilution. Subsequent incubation and washing steps were performed as outlined in the manufacturer’s protocol. In all analysed conditions detection of antibodies was performed using the FITC-labelled anti-human IgG solution supplied with the IIFT (EUROIMMUN; #FA1254-1) which was supplemented with Cy^TM^3-labelled donkey anti-rabbit IgG (H + L) (Jackson ImmunoResearch; #711-165-152) at a dilution of 1:200.

### In-house live cell-based assay (live CBA)

HEK cells were cultured in DMEM + 10% FCS + 1% antibiotics and grown until 80–100% confluent (37 °C, 8.5% CO_2_). Cells were disseminated onto reaction fields of a reagent tray for the indirect immunofluorescence test (= IIFT TITERPLANE Technique by EUROIMMUN). Cells were transfected with an expression plasmid encoding full-length recombinant human THSD7A. Preparation of sera and incubation with the HEK cells was performed as described for the IIFT. Reaction fields were covered with a slide against drying and the cells were incubated for 42 h at 37 °C and 8.5% CO_2_. Cells were washed with fresh DMEM and PBS plus 0.2% Tween-20.

## Supplementary Information

Supplementary Figures.
