# Supplementary material for: Characterization of THSD7A-antibodies not binding to glomerular THSD7A in a patient with diabetes mellitus but no membranous nephropathy
Source: Sci Rep. 2021 Aug 10;11:16188. doi: 10.1038/s41598-021-94921-y (PMC8355258; doi:10.1038/s41598-021-94921-y)
Supplement: Supplementary file 1 — Supplementary Figures. [file 41598_2021_94921_MOESM1_ESM.pdf]

Characterization of THSD7A-antibodies not binding to glomerular THSD7A in a patient with diabetes mellitus not having membranous nephropathy

Supplemental Information

Linda Reinhard[1], Cindy Thomas[2], Maya Machalitza[1], Erik Lattwein[2], Lothar S. Weiss[1], Jan Vitu[3], Thorsten Wiech[4], Rolf A.K. Stahl[1], Elion Hoxha[1*]

[1]: III. Department of Medicine, University Medical Center Hamburg-Eppendorf, Martinistrasse 52, 20246 Hamburg, Germany.

[2]: Institute of Experimental Immunology, EUROIMMUN AG, Lübeck, Germany.

[3]: Medizinisches Versorgungszentrum Hamburg-Sinstorf der MVZ gGmbH der PHV, Germany.

[4]: Institute of Pathology, Section Nephropathology, University Hospital Hamburg-Eppendorf, Martinistrasse 52, 20246 Hamburg, Germany.

*Correspondence:

Elion Hoxha, MD

III. Department of Medicine,

University Medical Center Hamburg-Eppendorf,

Martinistrasse 52, 20246 Hamburg, Germany

Email: ehoxha@uke.de

Supplemental Figures

Supplemental Figure 1: Full-lanes of Western blot analyses depicted in Figure 2.

In Western blot analyses serum of the patient was used as primary antibody, visualized with HRP-coupled anti-human IgG (unless otherwise indicated). For all three analyses, serum from a healthy donor served as negative control. Each depicted gel image presents one experiment, in which the membrane was cut for hybridisation with different antibodies and then re-assembled for chemiluminescent development. In the figure this is delineated by dividing lines. (a) The Western blots was performed under non-reducing, “standard” conditions (SDS present). No THSD7A-specific signal is detectable. For the positive control, serum from a THSD7A-antibody positive MN patient was used. (b) The Western blot was performed under non-reducing, “native” conditions (SDS absent) and did not give any detectable THSD7A-specific signal. For the positive control, serum from a THSD7A-antibody positive MN patient was used. (c) Western blot analysis under reducing, “standard” conditions (SDS present) gave a Western blot signal at the expected height for rTHSD7A, but not for HGE. For the positive control, rabbit anti-human THSD7A was used, visualized with HRP-coupled anti-rabbit IgG.

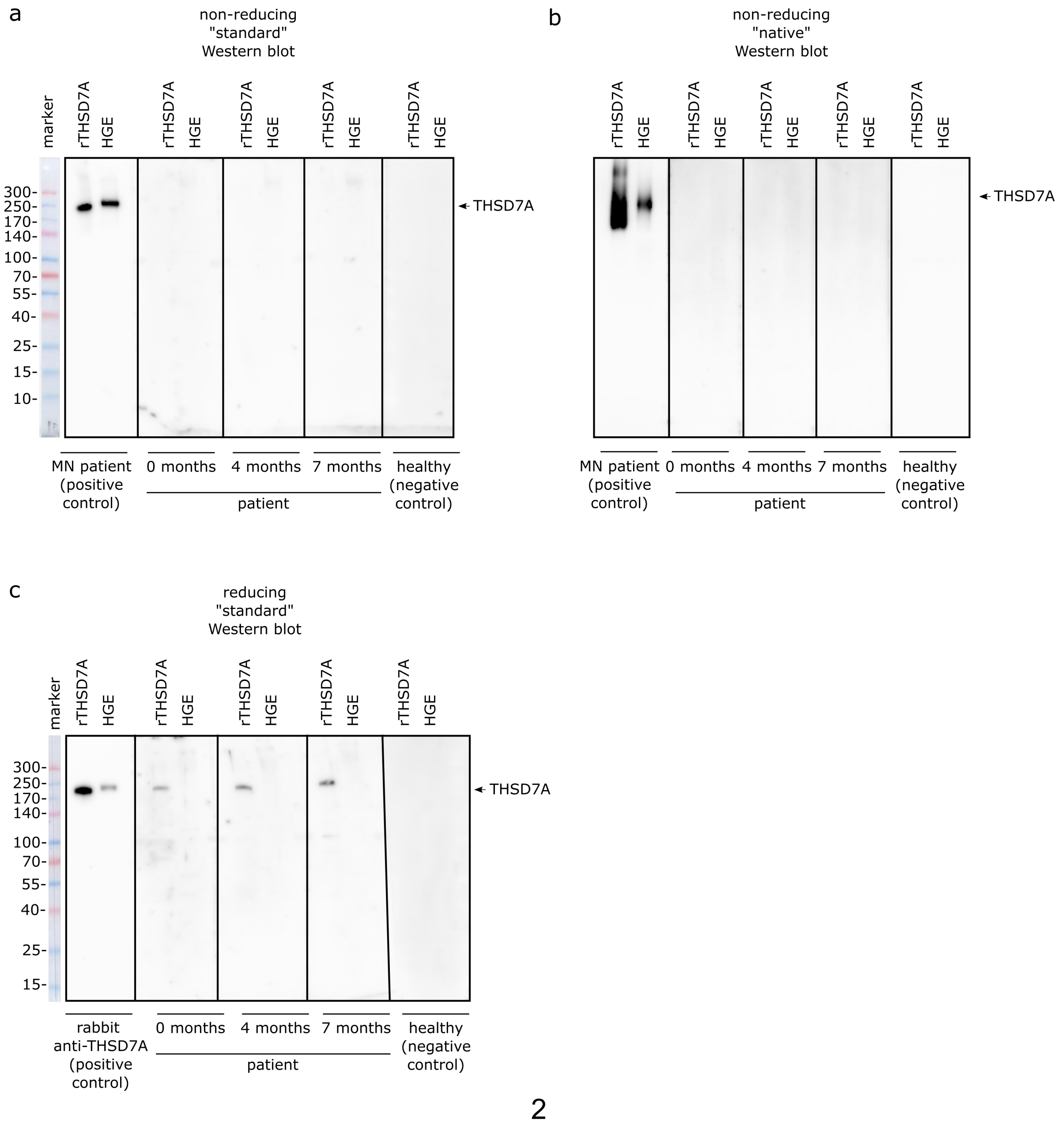

Supplemental Figure 2: IgG subclass analysis.

For the IgG subclass analyses, rTHSD7A was loaded as an antigen in the Western Blot analyses. As primary antibody, serum of the patients was used. The membrane was cut in marker lanes and membrane pieces were incubated in different anti-human IgG subclass antibodies. (a) IgG subclass analysis revealed that the THSD7A-specific Western blot signal obtained from the serum of the index patient under reducing conditions belongs to the IgG3 subclass. No other subclasses could be detected. (b-d) For three patients with THSD7A-associated MN, the antibodies belong to the IgG4 subclass, which is also the predominant IgG subclass detected by Western blot under non-reducing conditions in patients with THSD7A-associated MN. For MN patient 1, also a Western blot signal of the IgG3 subclass was detectable.

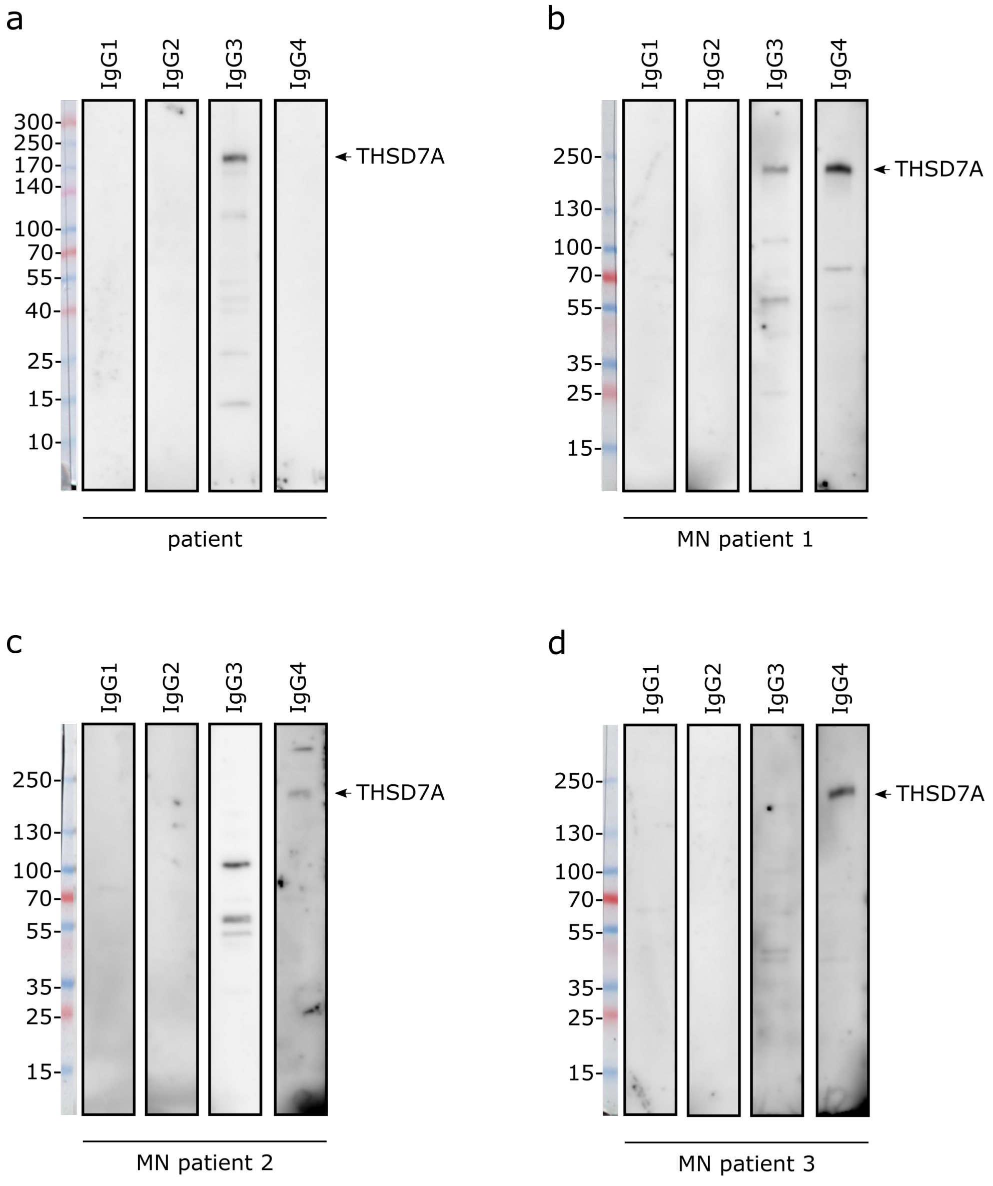

Supplemental Figure 3: Analysis of the glycosylation status.

(a) Whole cell lysate of rTHSD7A expressed in HEK293 cells was deglycosylated using three glycosidases (N-glycosidase F, O-glycosidase and Neuramidase) individually or in combination, respectively, and loaded as antigen in Western Blot analyses. The sera from the patient case (detected with HRP-coupled anti-human IgG3), a patient with THSD7A-associated MN (detected with HRP-coupled anti-human IgG4) and the rabbit anti-THSD7A control (detected with HRP-coupled anti-rabbit IgG) resulted in the same THSD7A recognition pattern. (b) Human IgG3 from the serum of the patient case, from a patient with THSD7A-associated MN and a healthy control loaded as antigens in the Western Blot analysis run at the same height in non-reducing, “standard” Western blot, showing that the IgG3 molecules exhibit the same size. No large differences in glycosylation patterns are observed.

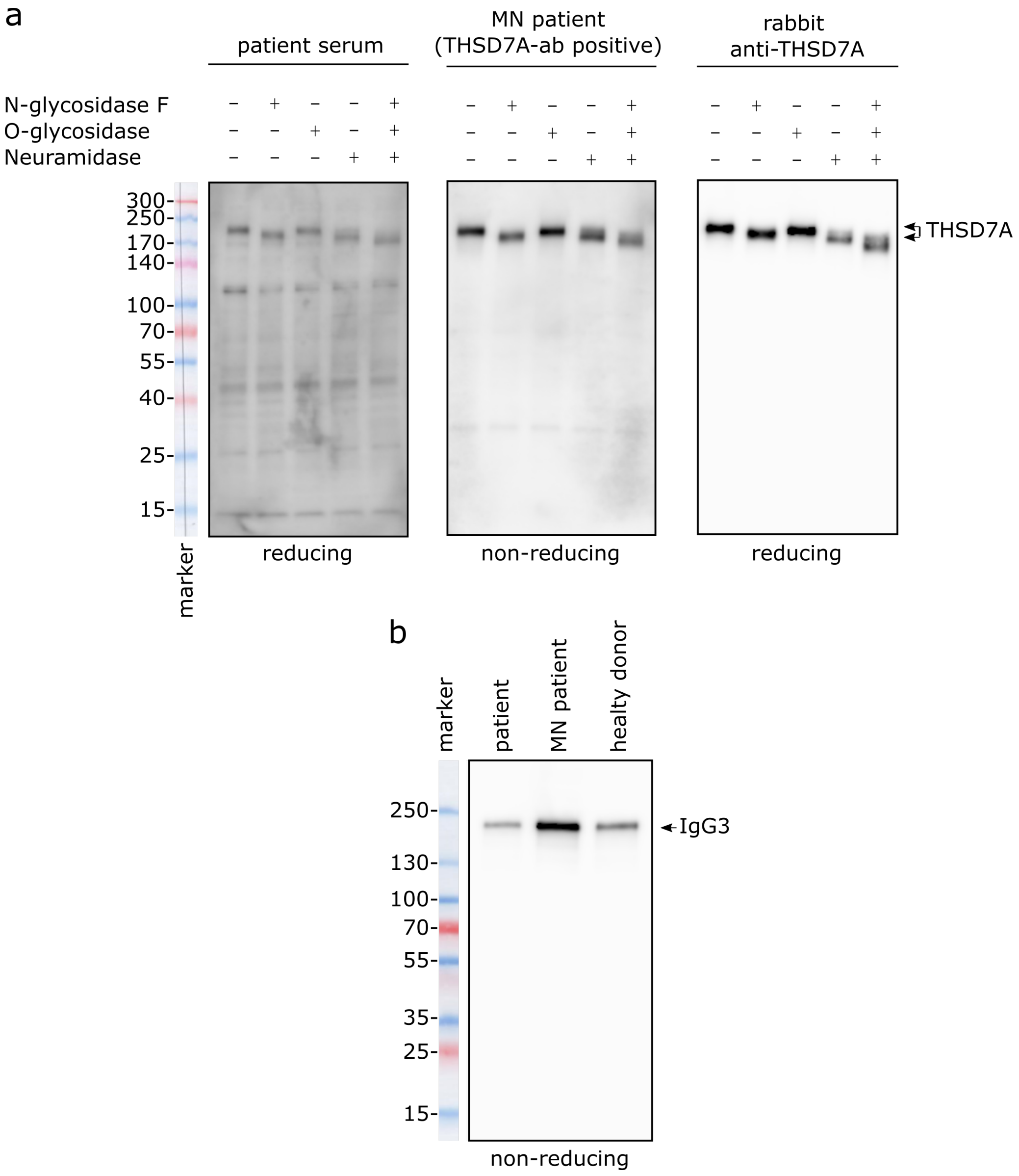

Supplemental Figure 4: Multiple exposure times of selected Western blot images.

Shown are three selected exposure times of the Western blot images presented in Supplemental Figure 4a (right) (a) and Supplemental Figure 4b (b).

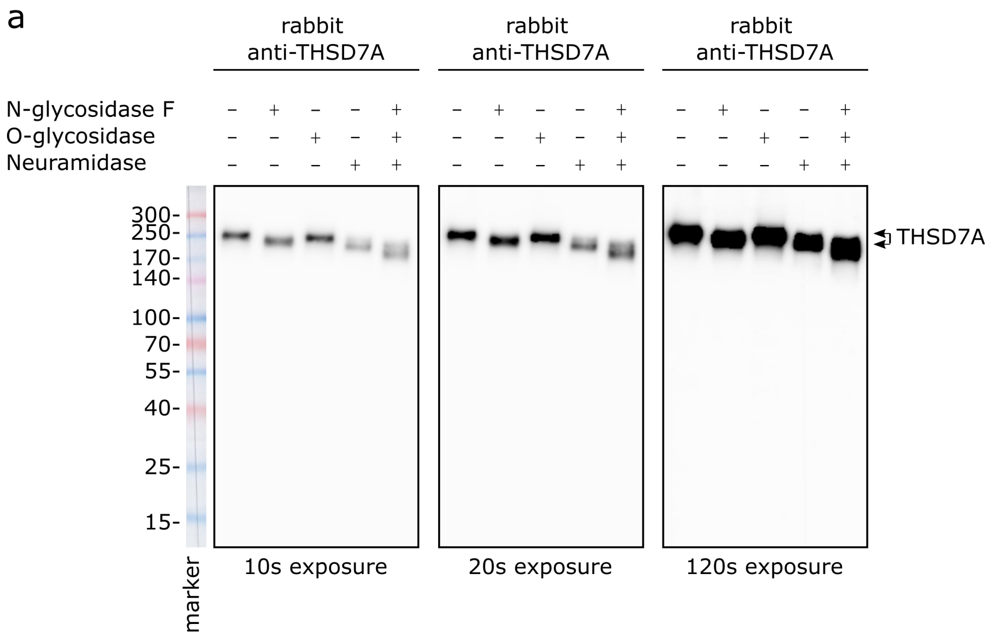

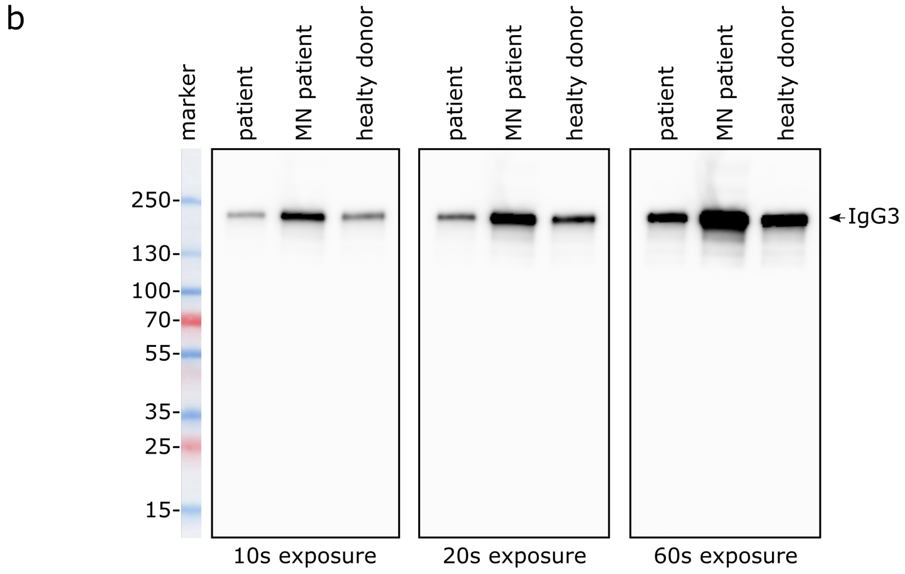

Supplemental Figure 5: Results of the live cell-based assay

(a) The patient serum, collected at the time of first presentation (0 months) does not show any detectable THSD7A-specific signal in the live cell-based assay. (b) As a positive control, the serum of a patient with THSD7A-associated MN was used, showing a distinct positivity for THSD7A.

a b

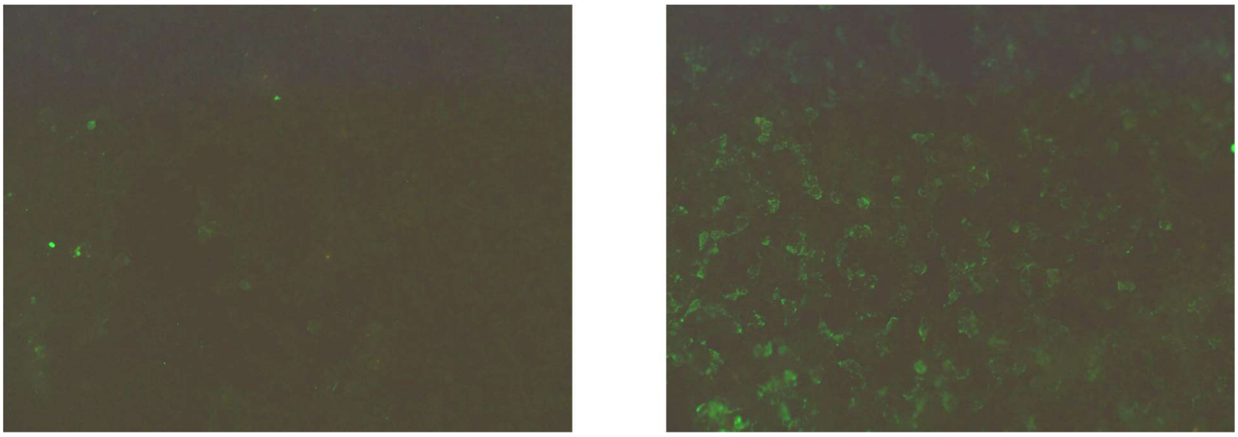

Index patient MN patient
